# Ovarian cancer resistance to PARPi and platinum-containing chemotherapy

**DOI:** 10.20517/cdr.2021.146

**Published:** 2022-06-22

**Authors:** Rebekah Summey, Denise Uyar

**Affiliations:** Department of Obstetrics and Gynecology, Division Gynecologic Oncology, Medical College of Wisconsin, Milwaukee, WI 53226, USA.

**Keywords:** Epithelial ovarian cancer, chemoresistance, platinum resistance, poly (ADP-ribose) polymerase inhibitor resistance, tumor microenvironment

## Abstract

Epithelial ovarian cancer remains the most lethal female malignancy despite options for systemic therapy and the emergence of targeted therapies. Although initial response to therapy is observed, recurrence and ultimately chemoresistance result in overall therapeutic failure. This pattern has been evident with platinum therapy since the 1980s. Significant excitement surrounded the approval of poly (ADP-ribose) polymerase inhibition (PARPi) as a novel therapeutic option, especially with the advent of personalized medicine, but resistance has similarly developed to these treatments. Novel agents are constantly being sought, but if the obstacle of chemoresistance remains, the durability of responses will remain tenuous. Unraveling the multifactorial mechanisms of platinum and PARPi resistance is increasingly important as a therapeutic failure with current strategies is almost assured. Focusing greater efforts on expanding the current understanding of the complex nature of platinum and PARPi chemoresistance has tremendous potential to improve clinical outcomes.

## EPITHELIAL OVARIAN CANCER AND THE LIMITATIONS OF CURRENT THERAPY 

Epithelial ovarian cancer (EOC), primarily high-grade serous carcinoma (HGSOC), remains the leading cause of death from gynecologic cancers primarily due to the advanced stage at the time of diagnosis and the inherent difficulty of treating tumors that become resistant to chemotherapy over varying amounts of time. Surgical cytoreduction and platinum-based chemotherapy have been the mainstay of ovarian cancer treatment since the 1980s. Platinum cytotoxicity results from its ability to cause an accumulation of deoxyribonucleic acid (DNA) double-strand breaks and cell death via the formation of intra-strand and inter-strand adducts which activate cellular DNA damage response (DDR) pathways, which inhibit replication and transcription of DNA, inducing cell cycle arrest and apoptosis^[1]^. Platinum-based therapy has been the cornerstone of EOC treatment for decades, but the majority of patients relapse within two years, and eventual platinum resistance is accompanied by an extremely poor prognosis^[2,3]^. Platinum-refractory disease is defined as a persistent or progressive disease while receiving platinum-based chemotherapy or within four weeks of therapy, and platinum resistance is defined as cancer recurrence or progression within six months of platinum therapy^[3]^. The median survival in platinum-resistant disease is only 12 months, underscoring the need to identify novel therapies but also to understand and overcome the complex mechanisms of chemoresistance^[4]^.

The advent of poly (ADP-ribose) polymerase inhibition (PARPi) as a novel therapeutic option approved for EOC in 2014 presented significant excitement and marked the birth of personalized medicine in EOC. Poly (ADP-ribose) polymerases (PARP) are a family of proteins with several roles that include DNA repair (including nucleotide excision repair, non-homologous end joining, homologous recombination (HR) and DNA mismatch repair), apoptosis, chromatin remodeling and the stress response^[5]^. Several clinical trials have demonstrated the impressive benefit of PARPi therapy, especially in patients who have responded to platinum-based therapy^[6,7]^. This might be attributable to the high prevalence of tumors with homologous recombination deficiency (HRD), which is found in approximately 50% of all ovarian cancers^[6]^. BReast CAncer gene (*BRCA1/2*) germline deficiency accounts for approximately 20% of these cases^[4]^. Somatic BRCA mutations result in HGSOC phenotypes and platinum chemotherapy responses similar to germline BRCA mutation carriers and are referred to as HGSOC with “BRCAness”. BRCA variants, either germline or somatic, lead to an increased susceptibility to DNA double-strand breaks. PARPi elegantly capitalizes on synthetic lethality when PARP inhibition and HRD are combined to cause apoptosis of cancer cells^[8]^.

Resistance to platinum therapy is strongly predictive of resistance to PARPi therapy consistent with overlapping biologic mechanisms of susceptibility and resistance^[2,9]^. A specific definition for PARPi resistance has not yet been developed as exists for platinum-based chemotherapy, and it remains to be seen if tumors are resistant to all PARPi once resistance is developed to a single PARPi^[6]^.

In this commentary, we will explain some of the most clinically relevant tumor biology contributing to platinum and PARPi resistance, two of the most significant therapies in use currently for EOC, as well as review future directions.

## MECHANISMS OF CHEMORESISTANCE

There are several mechanisms, both intrinsic and acquired, that contribute to platinum chemoresistance. Platinum compounds may undergo decreased uptake into cells, increased efflux, or increased inactivation. For example, overexpression of the adenosine triphosphate-binding cassette (ABC) family of transporters, such as P-glycoprotein or multidrug resistance-related proteins (MRP1), affects the efflux of chemotherapeutic drugs. Although the exact mechanism of action is not clear, high levels of ABC subfamily A member 1 expression have been found to correlate with poor progression-free and overall survival in serous EOC^[10]^. 

Once platinum cytotoxicity has occurred and adducts have begun to form, cells may still be able to thwart its effects by acquiring the ability to repair platinum-induced DNA adducts via counteraction of the cell’s DDR response. The DDR response is an essential network of proteins that sense, signal and/or repair DNA damage to maintain genomic integrity and stability within the cell^[11]^. Key proteins that signal DNA damage to cell-cycle checkpoints and DNA repair pathways include: ataxia-telangiectasia mutated (ATM), ATM-and RAD3-related, DNA-dependent protein kinases^[6]^. These key proteins sense DNA damage and initiate repair signaling cascades by phosphorylation of key repair proteins: BRCA1, checkpoint kinase 1 (CHK1), checkpoint kinase 2, tumor protein p53 (P53) and *RAD17*^[12]^. Cellular mechanisms that assist in the repair of DNA include base excision repair for single-strand breaks (SSBs); nucleotide excision repair (NER) for the repair of bulky adducts (such as observed with platinum therapy); mismatch repair (MMR) for mispaired bases; homologous recombination (HR) repair for double-stranded breaks (DSBs), intra-strand and inter-strand crosslinks; non-homologous end-joining (NHEJ) for DSB; or microhomology-mediated end joining for the repair of DSBs^[13]^. 

When DNA damage is too severe for repair, apoptosis is triggered^[13]^. Cells that have acquired platinum resistance subvert the normal DDR responses and instead demonstrate increased ability to initiate repair signaling, activate checkpoints to slow progression and allow repair, increase the pace of adduct repair or even develop the ability to tolerate DNA adducts without resorting to apoptosis^[1,14]^. 

The NER and HR repair pathways work very closely together. The NER pathway recognizes and removes bulky helix-distorting crosslink lesions via a step-wise process: damage recognition, unwinding of the DNA locally around damage, incision of damaged DNA by endonucleases, and DNA resynthesis and ligation^[15]^. This process allows NER to recognize DNA crosslinks and converts the crosslink to a DNA double-strand break. HR is then required to repair the double-strand break to prevent apoptosis^[16]^.

In preclinical trials, enhanced NER in ovarian cancer models has been associated with increased cisplatin resistance *in vitro*, supporting the importance of NER in the development of platinum chemoresistance^[17,18,19]^. Specifically, the excision repair cross-complementation group 1 (ERCC1) protein plays a key role in nucleotide excision repair. Dimerization of ERCC1 with xeroderma pigmentosum complementation group F (XPF) enables excision and repair of bulky cisplatin lesions in damaged DNA^[18]^. Overexpression of proteins in the NER pathway, including ERCC1, is associated with platinum resistance, likely via the ERCC1-XPF endonuclease^[19,20]^.

Additionally, P53 expression, common in HGSOC, promotes tumorigenesis as well as drug resistance via inhibition of apoptosis. Inhibited apoptosis contributes to proliferation and metastasis^[2]^. P53 expression specifically leads to growth phase 1 (G1) / synthesis (S) checkpoint deficiencies and cell-cycle dysregulation, subsequently placing greater stress on the growth phase 2 (G2) / mitosis (M) checkpoint for survival. Wee1 kinase inhibits activation of Cyclin-dependent kinases 1 (CDK1) and CDK2, making it a key cell cycle regulator of the G2/M transition. Alterations in Wee1 kinase may have implications for chemoresistance^[21]^. 

Although the majority of EOC responds to platinum therapy, even if only for a limited time, many do not exhibit any durable response to platinum therapy. Mechanisms of such intrinsic chemoresistance include proficient DNA repair, cyclin E1 (CCNE1) amplification, and mesenchymal or proliferative molecular subtypes. Approximately 20% of EOC cases have CCNE1 amplification, which are associated with primary resistance to platinum^[22]^.

The current understanding of the mechanism of PARPi resistance centers around Darwinian escape. Darwinian escape refers to an acquired secondary somatic mutation to restore HR in BRCA 1/2 (reversion mutations)^[7]^. BRCA reversion mutations have been identified in progression biopsies as well as cell-free DNA (cfDNA). cfDNA has shown polyclonality of multiple reversion mutations within a single patient when under pressure from treatment^[8]^. One mutation that has demonstrated restoration of DNA repair is *BRCA1-Δ11q*. Patients with *BRCA1-Δ11q* demonstrated decreased sensitivity to both cisplatin and PARPi^[23]^. BRCA reversion mutations have been identified in 13% of patients with platinum-refractory disease and 18% of patients with platinum-resistant disease^[5]^. Patients with BRCA reversion mutations experienced decreased progression-free survival when undergoing treatment with rucaparib^[24]^. The initial strength of response to PARPi has been shown to correlate with the severity of eventual tumor resistance^[5]^.

## TUMOR MICROENVIRONMENT AND METABOLISM IMPACT ON CHEMORESISTANCE 

Numerous studies have demonstrated the critical role of hypoxia in the tumor microenvironment and its association with platinum resistance via signaling pathways in DNA damage, mitochondrial activity, apoptosis autophagy, and drug efflux^[25,26]^. Chen *et al*.^[27] ^demonstrated that cisplatin-resistant cancer cells show increased intracellular hypoxia and decreased glucose uptake suggesting that platinum resistance might stem from alterations in glucose metabolism. Altered angiogenesis is another hallmark of intra-tumor vessels which also contributes to hypoxia, poor drug delivery, and chemoresistance^[28]^.

Cancer-associated fibroblasts, tumor-associated macrophages, and cancer stem cells have all been proposed influencers of prognosis and chemotherapy resistance. Stromal activation with extensive desmoplasia has been associated with poor clinical outcomes and acquired treatment resistance^[27,29]^. Platinum chemotherapy is also thought to influence anti-tumor immunity. Increased infiltration of immunosuppressive Cluster of Differentiation 163-positive macrophages and increased infiltration of regulatory forkhead box P3-positive T cells have favored tumor growth^[6,30-32]^. In contrast, the presence of tumor-infiltrating lymphocytes is positively correlated with survival^[6,33]^. Data on immune checkpoint therapy in EOC has been contradictorily reflecting an incomplete understanding of the key regulators and pathways^[6]^. Immunotherapy may yet play an important role in EOC.

Additionally, the omentum and cell metabolism are thought to play a pivotal role in cancer progression and chemoresistance. The omentum is a favored site for metastasis due to EOC’s ability to use fatty acids to initiate and sustain peritoneal metastasis; in addition, it may also participate in ascites formation^[34,35]^. Elevated cytokines and adipokines in the ascitic fluid are thought to protect malignant cells from chemotherapy-induced apoptosis, including via lysophosphatidic acid signaling and prostaglandin E2 -mediated GFTR transporter upregulation^[19]^. The metabolism of tumor cells is intricately intertwined with all cells of the microenvironment. Tumor cells have demonstrated an amazing ability to metabolically adjust to changes in their environment, as well as influence the metabolic function of neighboring cells for their own purposes^[34,35,36]^. 

Cancer stem cells (CSCs) are theorized as a population of cells capable of self-renewal and repopulation following cancer treatment that may cause tumor initiation and metastasis^[37]^. Stem cell pathways have recently been recognized as an important mediator of chemoresistance. CSCs have improved methods of chemotherapy removal from the cell, including the ABC cassette and drug transporters. Tumors containing heterogeneous cancer cells are linked to progression as they are more likely to undergo selection towards a population of drug-resistant tumor cells with treatment. CSCs are aided by their microenvironment and stimulated by hypoxia-inducible factors. Phosphoinositide kinase (PI3K) has an important role in apoptosis for maintaining stem cell “stemness” and drug resistance. Notch signaling and Wnt/beta-catenin are important in CSCs signaling pathways and may be useful targets^[38]^. CSCs are also able to grow in spheres and expand as sphere-like cellular aggregates more easily than other populations of cells^[39]^. The ability to treat cancer stem cells is of particular importance since these cells can remain in patients with seemingly no evidence of disease or lead to metastasis.

## FUTURE DIRECTIONS

Chemoresistance evolves over time as a response to the selective pressure of therapy. The multiple mechanisms of resistance are complex and not fully understood, but strategies to overcome chemoresistance are being developed. These methods include exploration of methods to improve drug delivery; identification of potentially useful biomarkers; identification of molecular targets; and exploitation of tumor weaknesses using modulation of the cell cycle, tumor microenvironment, or cellular metabolism. Combination therapies may improve this exploitation of tumor weaknesses.

### Drug delivery

Improved drug delivery could also combat chemoresistance. Nanotechnology is being used to investigate the ability of nanoparticles to improve targeted treatment, including the encapsulation of cisplatin with dendrimers to aid in cell killing^[2,40]^. Layer-by-layer nanoparticles have also been used to deliver cisplatin and a PARPi to mice, with improved bioavailability, cytotoxicity, and systemic toxicity compared with conventionally administered medication^[40]^. Natural compounds including curcumin and piperine are being evaluated to attempt to induce G2/M phase arrest, caspase activation, the PI3K pathway (piperine), and apoptosis and phosphorylation of p53 (curcumin)^[2]^. 

### Biomarkers

A study of patients with durable responses may be useful for identifying biomarkers, especially patients with HR proficiency who have good responses^[7]^. Improved understanding of why some patients with BRCA pathogenic variants do not respond to PARPi, and why some patients with no known BRCA mutation respond to PARPi treatment could be important for combatting drug resistance. Biomarkers that predict treatment success would be of significant benefit if they allowed improved treatment selection. However, our understanding of biomarkers that would be useful in guiding treatment selection is limited. A number of genes and proteins have been identified in patients with chemoresistance or poor prognosis and have yet to be evaluated in clinical trials to guide treatment. One gene amplification, 19q12, is associated with chemoresistance and has been identified in 20% of ovarian cancer patients^[3,40]^. MMR deficiency has also been implicated in chemoresistance, but the correlation is controversial^[3,19,41]^.

Based on the leading roles played by ERCC1 and XPF in the NER pathway, they have been promising prospects as biomarkers for platinum-based therapy. A meta-analysis of retrospective *in vitro* studies in non-small cell lung cancer by Altaha *et al*.^[42] ^concluded that high levels of ERCC1-mRNA and/or ERCC1 protein were associated with resistance to platinum compounds. Despite the correlation between ERCC1 overexpression with platinum resistance and ERCC1 under expression with platinum sensitivity, translating ERCC1 as a predictive biomarker for response to platinum-based therapy in clinical trials has been challenging^[43]^. ERCC1 has been investigated as a biomarker in both adrenocortical carcinomas and advanced non-small cell lung cancer and did not offer prognostic or treatment selection benefits^[44,45]^. Continued study of ERCC1 and the NER pathway is needed.

Patients with PARPi resistance have been found to have reversions in *RAD51C* and *RAD51D*, and in vitro studies have demonstrated loss of TP53 binding protein 1^[3,46]^. Studies of TP53 vary regarding its utility as a marker in platinum resistance, but it may be helpful in treatment planning^[47]^. Pump P-glycoprotein (P-gp) expression, which is related to drug efflux and multidrug resistance, has also been implicated in PARPi resistance^[3,48]^. CSC markers of *Bmi-1*, *Nanog* and *Oct 4* with elevated stem cell factor and *c-Kit* levels indicated chemoresistance as well^[37]^. Other proteins indicating resistance and poor prognosis include Notch3 and *LGR5*; CD24 correlates with poor prognosis, chemoresistance, metastasis, and recurrence; CD44+/CD117+ cells were associated with chemoresistance; CD133+ cells showed cisplatin resistance, and ALDH1A1 was associated with poor survival and drug resistance^[36,49]^. AT-rich interaction domain 1A inactivation or loss of expression is also correlated with poor overall survival in patients receiving platinum. Micro ribonucleic acids (miRNAs), including let-7 g, miR-98-5p, miR-622 as well as others, have been associated with platinum and/or PARPi resistance^[50]^.

Newer technology in tumor surveillance may also be critical to the understanding of tumor chemoresistance and the discovery of biomarkers. Liquid biopsies may offer a unique opportunity for real-time tumor molecular profiling. Liquid biopsies enable the analysis of circulating tumor cells, circulating tumor DNA, circulating mRNA, and tumor-derived extracellular vesicles that are shed from primary tumors into the peripheral blood^[51]^. Serial assessments would enable real-time patient assessment and comparison during therapy at the onset of resistance to therapy, which may increase our understanding of chemoresistance.

### Genetics

Other possible areas of investigation involve the utilization of next-generation sequencing to aid the discovery of predictive signatures. Four molecular subtypes of HGSOC have been described and validated by the Cancer Genome Atlas Research Network study^[52]^. Absolute copy number profiles have been generated using primary and relapsed HGSC samples. Macintyre *et al*.^[53] ^identified seven copy number signatures that were stable between diagnosis and disease relapse, indicating that copy number signatures at diagnosis may predict overall survival and platinum resistance^[54]^. Different mutational processes generate unique combinations of mutation types, termed mutational signatures^[55]^. The Catalogue of Somatic Mutations in Cancer (COSMIC) has revealed many mutational signatures across the spectrum of human cancer types. Analysis of COSMIC mutational signatures in ovarian cancer reveals a prevalence of signature 1B^[56]^.

Epigenetic modifications are mechanisms that alter the expression of a gene but do not change the DNA sequence. These modifications help to regulate normal genome functioning. Epigenetic modifications may also be contributing to chemoresistance. Identification of epigenetic changes in chemoresistant EOC may allow for the advanced development of epigenetic modulating drug therapy. Current agents such as demethylating agents, histone deacetylase inhibitors, and microRNA-targeting therapies have shown preclinical promise^[57]^.

### Homologous recombination and combination therapies

Defining PARPi resistance and evaluating the utility of alternating PARPi therapy if resistance has developed to one PARP remains to be seen but may be answered by the OReO trial^[6,58]^. PARPi do have different potencies due to the degree of PARP trapping; clinical implications have yet to be seen. Additionally, re-examining the definition of platinum sensitivity and resistance may be needed.

Combination therapies of PARPi and platinum together, or the addition of another agent to combat chemoresistance when using one or the other, may allow circumvention of treatment resistance. In a study by Gajan *et al*.^[58]^ of breast cancer patients with and without BRCA mutations and with resistance to both cisplatin and olaparib, a benefit of combined therapy with olaparib and cisplatin was identified. Interestingly, the combination treatment was not helpful in patients whose disease was still sensitive to either treatment^[59]^. A similar investigation could be considered in EOC patients as well. Niraparib has been investigated in combination with carboplatin and gemcitabine for patients with unresectable or recurrent platinum-sensitive ovarian cancer and showed a 57% radiographic response rate^[60]^. Cisplatin-resistant ovarian cancer cells (C13) were treated with a PARPi, 3-aminobenzamide by Zhang *et al.*^[60]^ Cellular proliferation was inhibited more when treated with cisplatin and increasing doses of PARPi, pointing to chemosensitization^[61]^. Additional studies investigating novel DDR-targeting agents and combination treatment approaches are underway.

As targetable pathways have been implicated in chemoresistance, translational researchers have also been searching for immunotherapies and molecular targets to use as combination therapies to improve the cytotoxic effect of therapy. Anti-angiogenesis with cediranib in combination with olaparib has been investigated with efficacy that varied by mutation identified following PARPi progression, with poorer outcomes identified for patients with HRD reversion mutations and ABCB1 upregulation^[62]^. This treatment combination is under further investigation with the OVC2 trial^[6,63]^. One promising target for therapeutics to combine with PARPi is mesenchymal-epithelial transition factor (c-Met), a receptor tyrosine kinase, since the high expression is associated with a poor prognosis. This biomarker and therapeutic target have been linked to PARP resistance and HR restoration^[7]^. CDK have also been proposed as targets since they control cell cycle progression and DNA damage control. Loss of CDK inhibition impairs BRCA function (BRCAness)^[7]^. PI3K inhibitors have been proposed as methods to extend the use of PARPi and are under investigation^[7,64,65]^. Other therapies that have been trialed include PARPi with alkylating agents, topoisomerase I inhibitors, WEE1 kinase inhibitors, PI3K inhibitors, radiation, immunotherapy, and DNA methyltransferase inhibitors. PARPi with topoisomerase I inhibitor allows inhibition of TOP1-PARylation, inhibition of homologous recombination and stimulation of NHEJ plus inhibition of tyrosyl-DNA-phosphodiesterase I. Clinical investigations of this combination have not been completed^[5]^. However, promising combination treatments to overcome PARPi or platinum resistance have not been identified.

## CONCLUSIONS

EOC is a disease marked by frequent relapses and responses to therapy before the eventual development of chemoresistance. Chemoresistance remains a steadfast obstacle to improving EOC survival. Platinum, the mainstay of EOC treatment, is limited by almost ubiquitous platinum resistance. While anti-angiogenic therapy and PARPi are promising new treatments, resistance has already been observed. The heterogeneity of EOC allows it to adapt in response to treatment and confounds attempts to identify clinically meaningful biomarkers and targets. Tumors constantly evolving with time and treatment seem to naturally develop resistance. 

In some cancers, real-time collection of circulating tumor DNA is a reality that has enabled the characterization of drug response for tumors along with multiple time points^[62]^. EOC, given its inherent heterogeneity, may require an equally malleable treatment approach: the serial integration of genomic, epigenomic, metabolomic, and immunogenic study at diagnosis and at each recurrence to optimize therapy. It would require a significant change in clinical practice and greater investment in bioinformatics, but it may not be possible to conquer all the nuances of EOC without it.

## DECLARATIONS

### Copyright

© The Author(s) 2022.
